# Research status and trends of physical activity on depression or anxiety: a bibliometric analysis

**DOI:** 10.3389/fnins.2024.1337739

**Published:** 2024-03-22

**Authors:** Xin-Yue Zhang, Fang Ye, Zi-Han Yin, Ya-Qin Li, Qiong-Nan Bao, Man-Ze Xia, Zheng-Hong Chen, Wan-Qi Zhong, Ke-Xin Wu, Jin Yao, Fan-Rong Liang

**Affiliations:** ^1^School of Acu-Mox and Tuina, Chengdu University of Traditional Chinese Medicine, Chengdu, Sichuan, China; ^2^Acupuncture Clinical Research Center of Sichuan Province, Chengdu, China; ^3^Department of Neurology, The Sichuan Province People's Hospital, Chengdu, China

**Keywords:** physical activity, depression, anxiety, bibliometric analysis, adult neurogenesis

## Abstract

**Background:**

Anxiety and depression are prevalent mental disorders. As modern society continues to face mounting pressures, the incidence of anxiety and depression is on the rise. In recent years, there has been an increasing breadth of research exploring the relationship between anxiety, depression, and physical activity (PA). However, the current research progress and future development trends are unclear. The purpose of this study is to explore the research hotspots and development trends in this field, and to provide guidance for future studies and to provide some reference for clinicians.

**Methods:**

We searched the relevant literature of Web of Science Core Collection from the establishment of the database to August 15, 2023. CiteSpace, VOSviewer and Bibliometrix Packages based on the R language were used to analyze the number of publications, countries, institutions, journals, authors, references, and keywords.

**Results:**

A total of 1,591 studies were included in the analysis, and the research in the field of PA on anxiety or depression has consistently expanded. The USA (304 publications), Harvard University (93 publications), and the journal of affective disorders (97 publications) were the countries, institutions, and journals that published the highest number of articles, respectively. According to the keywords, students and pregnant women, adult neurogenesis, and Tai Chi were the groups of concern, physiological and pathological mechanisms, and the type of PA of interest, respectively.

**Conclusion:**

The study of PA on anxiety or depression is experiencing ongoing expansion. Clinicians can consider advising patients to take mind–body exercise to improve mood. In addition, future researchers can explore the mind–body exercise and its impact on anxiety or depression, PA and anxiety or depression in specific populations, and adult neurogenesis of various exercise in anxiety or depression.

## Introduction

Mental disorders have emerged as a significant public health issue, among which depression and anxiety are the most prevalent and one of the primary causes of the global health burden (Santomauro et al., 2021). Indeed, they severely impact mental health, social function activities and quality of life. Depression is typically characterized by persistent low mood, loss of interest, cognitive impairment, sleep disorders, loss of appetite and suicidal tendencies (McCarron et al., 2021). The World Health Organization (WHO) has listed depression as the third leading cause of the global burden of disease (Malhi and Mann, 2018). Moreover, the previous study (Slavich and Irwin, 2014) reported that nearly 25% of women and 16% of men will experience depression, and over 50% of patients with depression will relapse. Anxiety disorders are chiefly manifested as episodic or persistent anxiety, tension or panic and other emotions (Rezaei et al., 2023), accounting for approximately 3% of the global burden of disease (Madonna et al., 2019). In a systematic retrospective study of disease prevalence studies in 44 countries, the global prevalence of anxiety disorders was estimated at 7.3% (Baxter et al., 2013). With the increasing pressure of modern human society, the accelerated pace of life is accompanied by an increase in a variety of various stress factors, leading to an increase in the incidence of depression and anxiety disorders, becoming one of the world’s major concerns.

The pathogenesis of depression and anxiety disorders remains unclear despite considerable research in the field (Malhi and Mann, 2018) involving the regulation of multi-system dysfunction. Through extensive clinical and animal experiments, genetics, biology, and social psychology have been established to participate in the development of diseases (Guilloux et al., 2011; Beesdo-Baum and Knappe, 2012; Fischer et al., 2018; Malhi and Mann, 2018). Drug therapy is a common clinical approach but is associated with side effects. Antidepressants have potential adverse reactions such as gastrointestinal symptoms, respiratory distress, hepatotoxicity, and allergic reactions (Carvalho et al., 2016). On the other hand, anxiolytics can lead to addiction (Offidani et al., 2013), gastrointestinal disorders (Harvey et al., 2000), and sexual dysfunction (Clayton et al., 2002). However, physical activity (PA) is considered a critical health behavior. The term “physical activity intervention” captures a broad range of interventions aimed at increasing energy expenditure above resting levels (Caspersen et al., 1985), encompassing aerobic exercise, resistance exercise, weight training, dance, yoga, tai chi, wuqinxi, baduanjin, and yijinjing (Noone et al., 2018).

In the previous studies, researchers focused on the health benefits of PA and revealed that exercise can mitigate the risk of diseases such as cardiovascular disease (Valenzuela et al., 2023), obesity (Hao et al., 2023) and diabetes (Kazeminasab et al., 2023). Afterward, studies have shifted toward the relationship between exercise and mental health. The 2018 American Sports Guide Advisory Committee report highlighted that PA can alleviate anxiety and depression symptoms (Pate, 2019). Previous evidence-based studies have also established that PA is an effective, safe, low-cost, and convenient method for the treatment of depression and anxiety (Mammen and Faulkner, 2013; Singh et al., 2023). Likewise, Singh et al. (2023) demonstrated that physical activity can significantly relieve symptoms of depression, anxiety and distress across a wide range of adult populations. Kvam et al. (2016) found that physical exercise is an effective intervention for depression. However, the current research progress and future development trends are unclear, impeding further exploration and development of PA interventions for anxiety and depression.

Bibliometrics is a research method commonly adopted to analyze scientific literature and identify research hotspots and emerging trends (Chen et al., 2012). The advantage of bibliometrics analysis lies in the wide range of included studies. Through the intuitive visual display of bibliometrics, the development of a specific field can be more intuitively, comprehensively, and systematically elucidated. Consequently, this research method has been widely applied across various fields (Chen et al., 2012; Dong et al., 2021; Cui et al., 2023). In the previous searches, relevant bibliometrics analysis were identified but were limited in terms of time (You et al., 2021), population (Ai et al., 2023) and disease type (Zhai and Xu, 2023). To the best of our knowledge, there is no comprehensive bibliometric analysis addressing on the current status and trends of PA on anxiety and depression. Therefore, the objective of this study is to present the current research status, hotspots, and trends of PA on anxiety and depression, and to lay a theoretical reference for subsequent research directions and to help clinicians better carry out clinical work.

## Methods

To date, there are no standardized guidelines for bibliometric analysis. Nevertheless, this study was conducted in accordance with the recommendations of the Preferred Reporting Items for Systematic Reviews and Meta-Analyses 2020 (Page et al., 2021) (PRISMA 2020) (Appendix 1). Additionally, this study was registered on the Open Science Framework (OSF) with the registration DOI: https://doi.org/10.17605/OSF.IO/7NJUP.Registration after data analysis was selected based on specific circumstances.

### Data collection and search strategies

In this study, the relevant literatures of Web of Science Core Collection (WoSCC) from the establishment of the database to August 15, 2023 were searched. Because we only focus on research trends and hotspots, based on previous bibliometric studies (Xiong et al., 2021; Mendlowicz et al., 2022; Yang et al., 2022; Ji et al., 2023), articles were screened by title rather than topic in order to avoid the influence of confounding factors in the abstract. The WoSCC search formula was set as follows: TI = [(‘depressive disorder’ OR ‘depression’ OR ‘anxiety’) AND (‘exercise’ OR ‘training’ OR ‘dance’ OR ‘aerobic’ OR ‘fitness’ OR ‘cardio ‘OR ‘physical activity’ OR ‘resistance exercise’ OR ‘weight training’ OR ‘yoga’ OR ‘taichi’ OR ‘wuqinxi’ OR ‘baduanjin’ OR ‘yijinjing’)]. The article retrieval and data extraction were completed within 1 day on August 15, 2023, to avoid potential biases caused by daily database updates.

**Figure 1:**
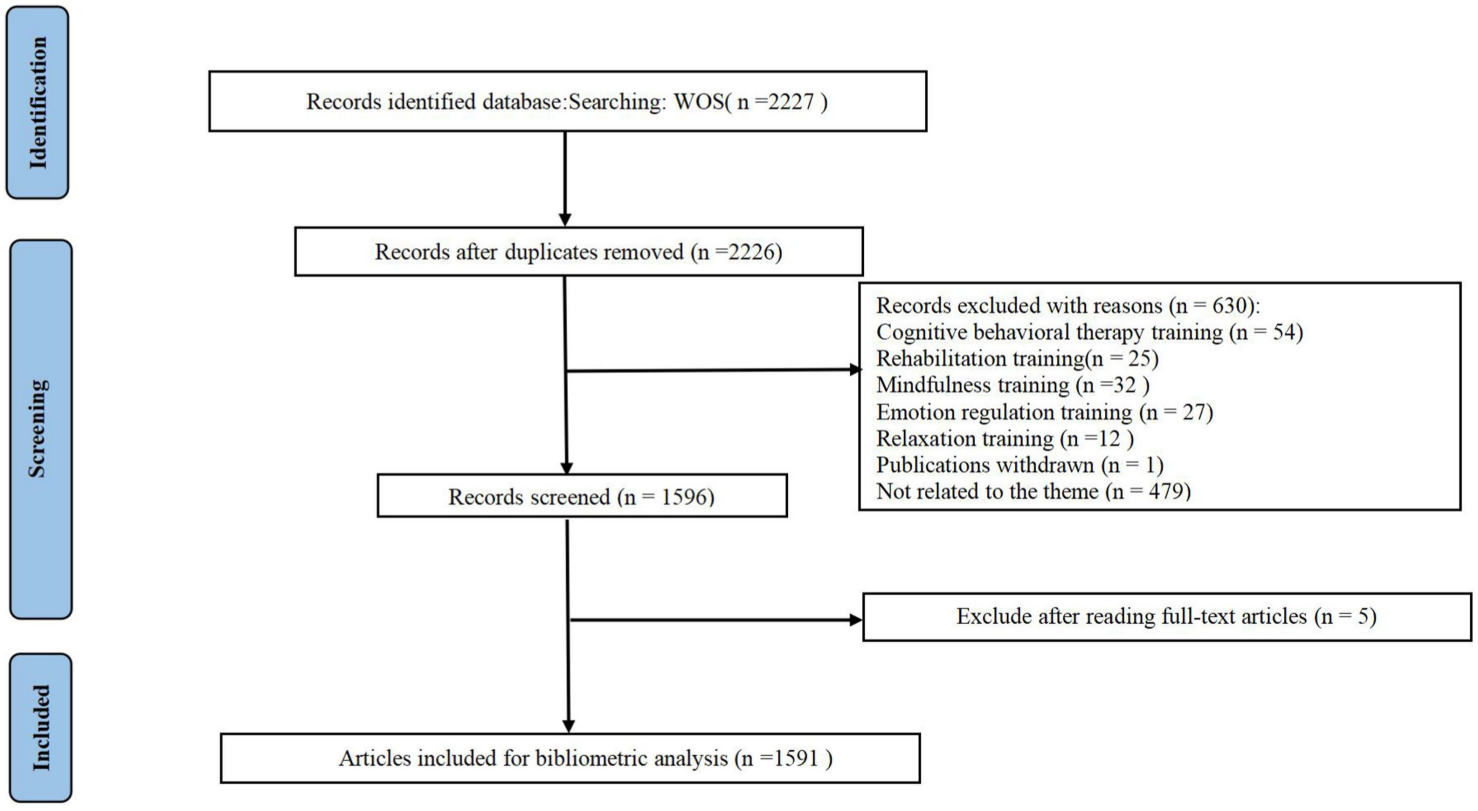
The detailed retrieval process.

Two independent reviewers (X-YZ and Y-QL) screened titles, keywords, and abstracts for relevance. Duplicate and ineligible studies were excluded. All remaining studies were examined for inclusion once the full text had been reviewed. In case of disagreements, a third reviewer (F-RL) counterchecked and arbitrated. The detailed retrieval process was shown in Figure 1.

### Inclusion/ exclusion criteria

Inclusion criteria were: (1) Peer-reviewed published articles on physical activity and anxiety or depression, including basic and clinical research; (2) Physical activities include aerobic exercise, resistance exercise, weight training, dance, yoga, tai chi, wuqinxi, baduanjin, and yijinjing; (3) The main types of literatures chosen for our study were articles and reviews; (4) The language of the article is limited to English.

Exclusion criteria were: (1) The articles unrelated to the research theme; (2) Articles not officially published; (3) Conference abstracts and proceedings and corrigendum documents; (4) Articles not in English language.

### Statistical analysis and tools

In this study, CiteSpace (version 6.2.4), VOSviewer (version 1.6.19), and Bibliometrix Packages based on the R language (version 4.3.1) were used to perform this bibliometric analysis.

CiteSpace is mainly used to count centrality and generate cooperation network visualizations. It can reveal changes in emerging trends, identify research frontiers (Chen et al., 2012). The visualization map consists of nodes and lines, where the size of the nodes is determined by the number of items (Chen and Song, 2019), and the connections between the nodes reveal collaboration or references. The betweenness centrality measure quantifies the importance and connectivity of the node’s location in the network. The higher the betweenness centrality is, the more the related connections are (Zhu et al., 2022). The basic parameters were as follows: time slicing (2011–2023), years per slice (5), top N per slice (12), pruning (pathfinder, pruning sliced networks and pruning the merged network).

Vosviewer is an analysis software based on network data to construct a visual network map (van Eck and Waltman, 2010), which can systematically understand the structure and dynamic development of scientific research. In Vosviewer’s cooperation network visualizations map and co-citation network visualizations map, different colors represent different clusters, and the lines between circles represent the cooperative relationship between different points. In this study, VOSviewer 1.6.18 was used to visualize the authors and references based on data.

**Figure 2:**
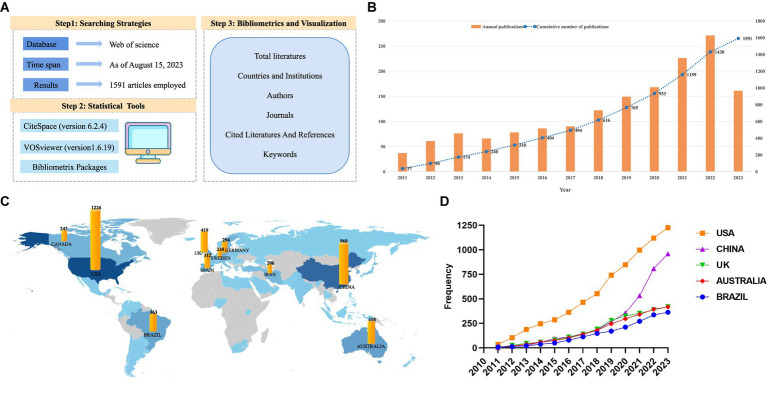
**(A)** The detailed method. **(B)** The number of articles published and the annual cumulative number of publications in each year about PA on depression or anxiety. **(C)** The top 10 countries by publication frequency. **(D)** The annual publication frequency line chart of the top 5 countries.

The bibliometrix is an online visualization package based on R studio. Literature analysis statistics, index calculation, network analysis and knowledge map drawing can be carried out. The detailed method is shown in Figure 2A.

## Results

### Literature screening results

According to the literature search strategy, a total of 2,227 articles were retrieved. To read the titles, abstracts, and even the full texts of paper to filter out papers that are closely related to our subject. Only 1,591 publications were enrolled. The main information of the included studies is shown in Table 1.

**Table 1:** The main information of the included studies.

Main information about data	Results
Timespan	2011:2023
Articles	1,591
Annual Growth Rate %	13.04
Article Average Age	4.19
Average Citations Per Article	25.79
Journals	590
Institutions	2,362
Countries	78
Authors	7,639
Authors of Single-authored Articles	27
References	54,542

### Publication growth and outputs

The change in the number of papers published annually is an important indicator, and the number of articles published during each period provides an intuitive indication of research trends in this field. According to our search strategy, the publication time of the 1,591 articles included was statistically analyzed, exposing that the number of related articles progressively increased over time (Figure 2B). Given that the retrieval time extended up to August 15,2023, the number of publications in 2023 currently appeared to be lower than that in 2022. However, the annual cumulative number of publications exhibited a consistent upward trend. According to the growth curve of the number of published papers, we postulate that the impact of PA on anxiety or depression is garnering increasing attention.

### Analysis of countries and institutions

A total of 78 countries participated in studies investigating the effect of PA on depression or anxiety. According to Table 2, the USA (1,226 times) was the leading country in terms of related publications, followed by China (960 times) and the UK (419 times) (Figure 2C). As illustrated in Figure 2D, the frequency of studies published in the United States and China has significantly increased on a yearly basis. Moreover, according to the national statistics of the corresponding author in the article, the USA emerged as leading country (304 publications), followed by China (280 publications) and Brazil (87 publications). The multiple country publications ratio refers to the proportion of co-authored articles in a country’s published articles. A larger value reflects a higher number of articles and a higher degree of cooperation with other countries. Interestingly, the MCP ratio value of Brazil was the largest (0.448), indicating that the country extensively cooperated with other countries. In addition, Brazil and Sweden had the highest centrality (over 0.1), implying that these two countries are the primary research centers in the field of the role of PA on depression or anxiety.

**Table 2:** Top 10 countries by frequency, centrality, articles and MCP_Ratio.

Rank	Country	Frequency	Rank	Country	Centrality	Rank	Country	Articles^a^	Rank	Country	MCP_Ratio^b^	Rank
1	USA	1,226	1	BRAZIL	0.61	1	USA	304	1	BRAZIL	0.448	1
2	CHINA	960	2	SWEDEN	0.26	2	CHINA	280	2	SPAIN	0.371	2
3	UK	419	3	CHINA	0.05	3	BRAZIL	87	3	UK	0.333	3
4	AUSTRALIA	418	4	SPAIN	0.05	4	AUSTRALIA	86	4	GERMANY	0.329	4
5	BRAZIL	363	5	USA	0.05	5	GERMANY	70	5	AUSTRALIA	0.291	5
6	SPAIN	312	6	AUSTRALIA	0.05	6	UK	66	6	CANADA	0.291	6
7	GERMANY	294	7	UK	0.00	7	SPAIN	62	7	IRAN	0.273	7
8	CANADA	243	8	GERMANY	0.00	8	KOREA	61	8	CHINA	0.218	8
9	SWEDEN	230	9	CANADA	0.00	9	CANADA	55	9	USA	0.122	9
10	IRAN	206	10	IRAN	0.00	10	IRAN	55	10	KOREA	0.082	10

a Articles, number of articles in national statistics by corresponding author.

b Multiple country publications ratio, refers to the proportion of co-authored articles in a country’s published articles.

A total of 2,362 institutions published articles of PA on depression or anxiety. As summarized in Table 3, among the top ten institutions, two institutions were from the USA and the UK, respectively. Notably, the top three high-yield institutions in this field were *HARVARD UNIVERSITY* (93 publications), *UNIVERSITY OF LONDON* (75 publications) and *KING ‘S COLLEGE LONDON* (69 publications). While the publication volume of *KU LEUVEN* was not the highest, its centrality was the highest (0.66), indicating that the organization plays an important role in the field. In addition, the centrality of *UNIVERSITY OF LONDON* (0.32), *KING ‘S COLLEGE LONDON* (0.37), and *KAROLINSKA INSTITUTET* (0.47) was also greater than 0.1, signifying that they also had a strong academic influence in this field. From the aforementioned results, it can be deduced that in the field of PA with depression or anxiety, despite the USA and China having published a large number of papers, their influence and cooperation with other countries remain sub-optimal.

**Table 3:** Top 10 institutions distributed by publications and centrality.

Rank	Institutions	Publications	Centrality	First publication year	Original country
1	HARVARD UNIVERSITY	93	0.08	2011	USA
2	UNIVERSITY OF LONDON	75	0.32	2012	UK
3	KING’S COLLEGE LONDON	69	0.37	2016	UK
4	KAROLINSKA INSTITUTET	64	0.47	2013	Sweden
5	KU LEUVEN	59	0.66	2013	Belgium
6	VRIJE UNIVERSITEIT AMSTERDAM	55	0.02	2016	Netherlands
7	UNIVERSITY OF CALIFORNIA SYSTEM	52	0.09	2012	USA
8	CHONGQING MEDICAL UNIVERSITY	48	0.00	2016	China
9	UNIVERSITY OF BASEL	47	0.00	2016	Switzerland
10	UNIVERSITY OF COPENHAGEN	46	0.04	2011	Denmark

### Analysis of authors

**Figure 3:**
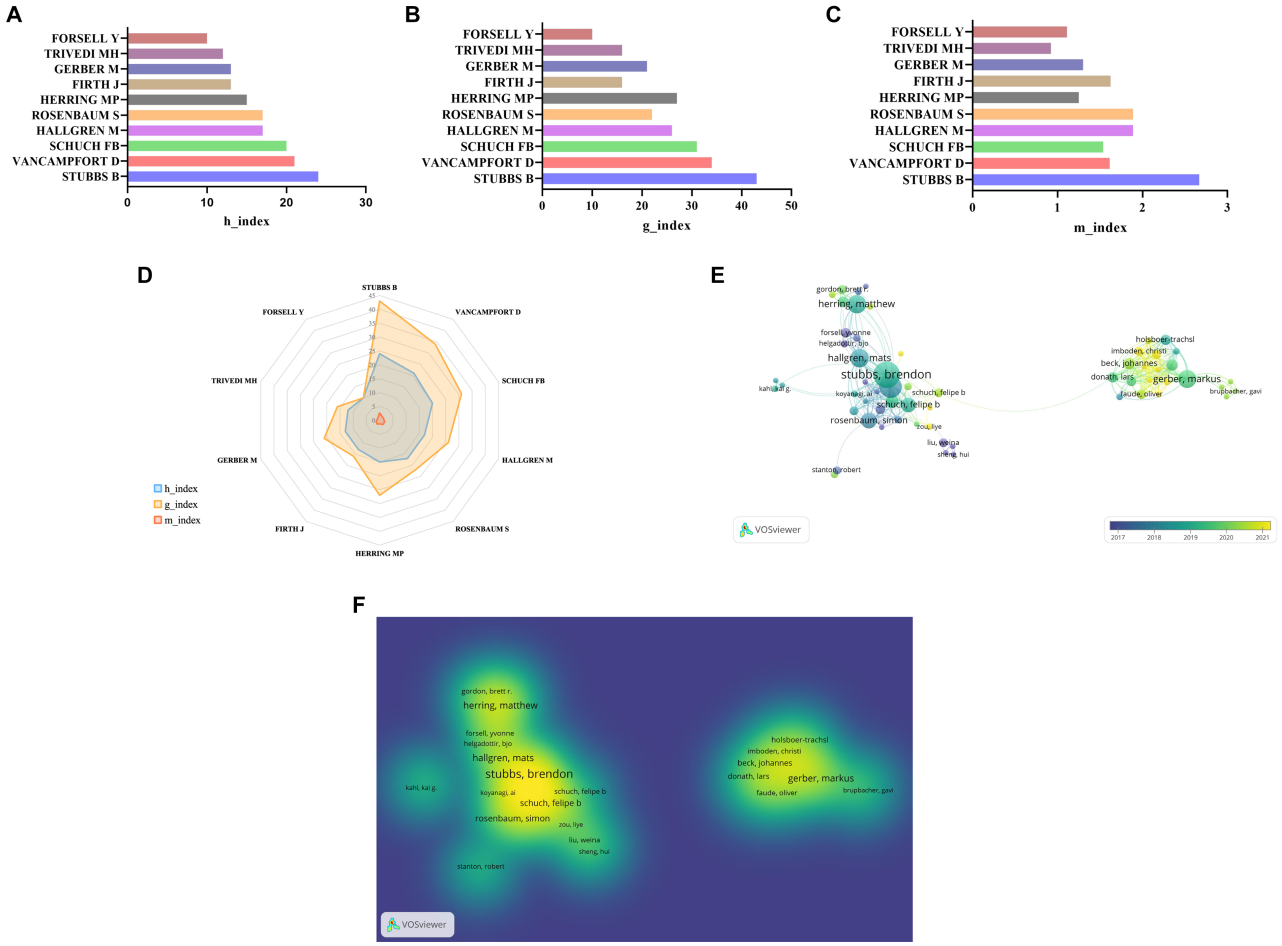
**(A)** Top 10 authors in terms of h-index. **(B)** Top 10 authors in terms of g-index. **(C)** Top 10 authors in terms of m-index. **(D)** The radar map of the top 10 authors in terms of h-index, g-index and m-index. **(E)** The author’s network visualization. **(F)** The author’s density visualization.

According to the results, a total of 7,639 authors published articles of PA on depression or anxiety. As detailed in Table 4, the top three authors in terms of the H-index were Stubbs B (24), Vancampfort D (21), and Schuch FB (20), with these authors also leading in total citations. Besides, the number of publications of these three authors was 43, 34, and 31, respectively. The H-index is a standardized indicator that reflects the number and impact of publications at the individual level (Hirsch, 2005; Hirsch, 2007). Of note, the g-index and m-index were derived from the h-index (Ali, 2021). These three indexes can collectively reflect the individual’s academic influence. As displayed in Figures 3A–D, Stubbs B, from the King’s College London in the UK, was the most prominent author, ranking first in all three indices. Although Stubbs B’s study of PA on depression or anxiety was initiated in 2015, it ranked first in terms of total citations. According to the ranking of the H-index, two of the top ten authors originated from the UK, demonstrating that the influence of the UK in this field should not be underestimated. According to the author’s network visualization (Figure 3E) and density visualization (Figure 3F), a cooperative network centered on Stubbs B and Gerber M was formed, wherein the former had more and closer cooperation. However, cooperation between the two authors is still lacking.

**Table 4:** Top 10 authors distributed by publications.

Authors	H_index	G_index	M_index	TC^a^	NP^b^	PY_start^c^	Institutions	Country
STUBBS B	24	43	2.667	4,521	43	2015	King’s College London	UK
VANCAMPFORT D	21	34	1.615	4,085	34	2011	Ku Leuven	Belgium
SCHUCH FB	20	31	1.538	3,629	31	2011	Federal University of Santa Maria	Brazil
HALLGREN M	17	26	1.889	1935	26	2015	Karolinska Institutet	Sweden
ROSENBAUM S	17	22	1.889	3,652	22	2015	University of New South Wales	Australia
HERRING MP	15	27	1.250	748	30	2012	University of Limerick	Ireland
FIRTH J	13	16	1.625	2,414	16	2016	University of Manchester	UK
GERBER M	13	21	1.300	445	24	2014	University of Bern	Switzerland
TRIVEDI MH	12	16	0.923	669	16	2011	University of Texas Southwestern Medical Center	USA
FORSELL Y	10	10	1.111	419	10	2015	Karolinska Institutet	Sweden

a TC,Total citation.

b NP, The numberof publications.

c PY stat, The year for the first pubication.

### Analysis of journals

A total of 590 journals published relevant articles of PA on depression or anxiety. According to the number of publications (Table 5), the top three magazines are *JOURNAL OF AFFECTIVE DISORDERS* (97 publication), *INTERNATIONAL JOURNAL OF ENVIRONMENTAL RESEARCH AND PUBLIC HEALTH* (73 publication), and *FRONTIERS IN PSYCHIATRY* (48 publication) respectively. The top 10 most productive journals in Table 4 are mainly in Q1 or Q2. The top three journals of H-index in this field are *PLOS ONE* (268, IF3.7), *JOURNAL OF AFFECTIVE DISORDERS* (165, IF6.6), and *BEHAVIORAL BRAIN RESEARCH* (154, IF11.3).

**Table 5:** Top 10 journals distributed by publications.

Rank	Sources	Publications	JIF quartile	IF(JCR 2023)	h-index	Publishers	Country
1	JOURNAL OF AFFECTIVE DISORDERS	97	Q1	6.6	165	Elsevier	Netherlands
2	INTERNATIONAL JOURNAL OF ENVIRONMENTAL RESEARCH AND PUBLIC HEALTH	73	/	/	78	MDPI	Switzerland
3	FRONTIERS IN PSYCHIATRY	48	Q2	4.7	52	Frontiers Media S.A.	USA
4	MENTAL HEALTH AND PHYSICAL ACTIVITY	26	Q2	4.7	33	Elsevier	Netherlands
5	FRONTIERS IN PSYCHOLOGY	25	Q1	3.8	110	Frontiers Media S.A.	Switzerland
6	PSYCHIATRY RESEARCH	21	Q1	11.3	118	Elsevier	Netherlands
7	BEHAVIORAL BRAIN RESEARCH	19	Q2	2.7	154	Elsevier	Netherlands
8	BMC PUBLIC HEALTH	19	Q2	4.5	117	BioMed Central	UK
9	DEPRESSION AND ANXIETY	19	Q1	7.4	110	Wiley-Blackwell Publishing Ltd	USA
10	PLOS ONE	19	Q2	3.7	268	Public Library of Science	USA

### Analysis of cited literatures and references

The top ten highly cited articles in the field of PA on depression or anxiety based on total citations are listed in Table 6. The top three were articles published by Stanton R in 2020 (Stanton et al., 2020) (740 citations), followed by articles published by Schuch et al. (2016) (709 citations), titled “Exercise as a treatment for depression: A meta-analysis adjusting for publication bias,” and Cooney GM in 2013 (Cooney et al., 2013) (661 citations), titled “Exercise for depression.” Despite the article published by Stanton R in 2020 (Stanton et al., 2020) being the most recent one, it garnered the highest number of citations. This observation may be attributed to the increased attention paid to the novel coronavirus in recent years. This paper (Stanton et al., 2020) largely examined the relationship between depression, anxiety and physical activity of adults during the COVID-19 pandemic.

**Table 6:** Top 10 cited literatures.

Rank	Title	Total Citations	Authors	Source	Year	DOI
1	Depression, Anxiety and Stress during COVID-19: Associations with Changes in Physical Activity, Sleep, Tobacco and Alcohol Use in Australian Adults	740	Stanton R	Int J Een Res Pub He	2020	10.3390/ijerph17114065
2	Exercise as a treatment for depression: A meta-analysis adjusting for publication bias	709	Schuch FB	J Psychiatr Res	2016	10.1016/j.jpsychires.2016.02.023
3	Exercise for depression	661	Cooney GM	Cochrane Db Syst Rev	2013	10.1002/14651858.CD004366.pub6
4	Physical activity and the prevention of depression: a systematic review of prospective studies	646	Mammen G	Am J prev Med	2013	10.1016/j.amepre.2013.08.001
5	Physical Activity and Incident Depression: A Meta-Analysis of Prospective Cohort Studies	643	Schuch FB	Am J Psychiat	2018	10.1176/appi.ajp.2018.17111194
6	Sedentary behavior and physical activity levels in people with schizophrenia, bipolar disorder and major depressive disorder: a global systematic review and meta-analysis	446	Vancampfort D	World Psychiatry	2017	10.1002/wps.20458
7	Exercise as a treatment for depression: A meta-analysis	409	Kvam S	J Affect Disorders	2016	10.1016/j.jad.2016.03.063
8	Exercise for the treatment of depression and anxiety	393	Carek PJ	Int J Psychiat Med	2011	10.2190/PM.41.1.c
9	A review of lifestyle factors that contribute to important pathways associated with major depression: diet, sleep and exercise	366	Lopresti AL	J Affect Disorders	2013	10.1016/j.jad.2013.01.014
10	Physical activity and depression: Towards understanding the antidepressant mechanisms of physical activity	330	Kandola A	Neurosct Biobehav R	2019	10.1016/j.neubiorev.2019.09.040

**Figure 4:**
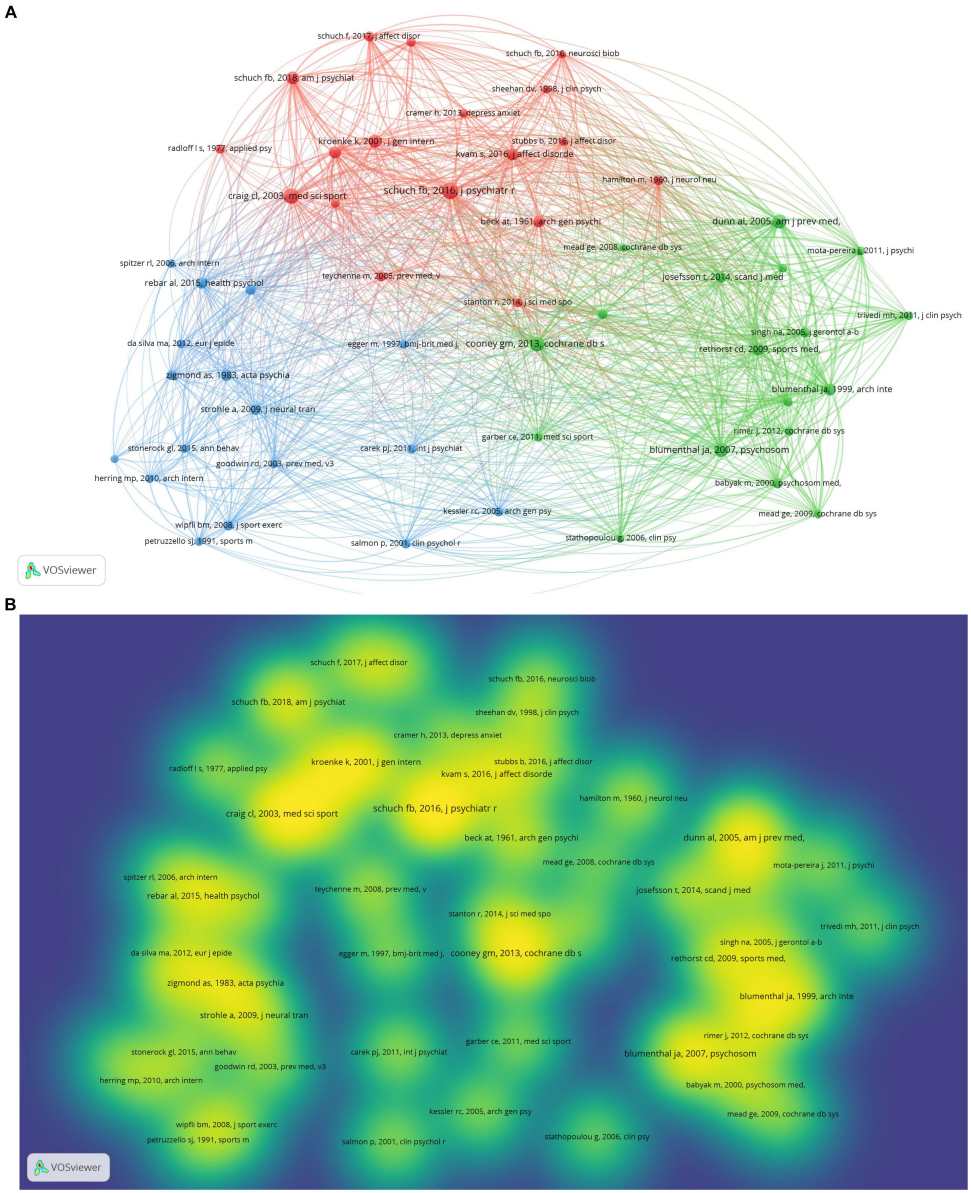
**(A)** The Cited references network visualization. **(B)** The Cited references density visualization.

The final analysis results involved a total of 54,542 references. As portrayed in Table 7, based on the strength of the total link strength (TSL), the top three were those published by Schuch et al. (2016) (TLS 760), Dunn Al in 2005 (Dunn et al., 2005) (TLS 623), and Blumenthal Ja in 2007 (Blumenthal et al., 2007) (TLS 616). They were related to exercise and depression. Research with a larger number of citations holds considerable implications in this field and offers a theoretical foundation for subsequent research by others. Using Vosviewer, the minimum number of citations of cited references was 40, and the cited references network visualization (Figure 4A) and density visualization were generated (Figure 4B). As delineated in visualization map, the three clusters are closely related. Cluster 1was based on Schuch Fb’s article published in 2016 (Schuch et al., 2016) (TLS 760), cluster 2 was based on Dunn Al ‘s article published in 2005 (Dunn et al., 2005) (TLS 623), whilst cluster 3 was based on Rebar AL’s article published in 2015 (Rebar et al., 2015) (TLS 267). We further analyzed the meta-analysis studies in cited literatures and references (Table 8). However, from these eight meta-analyses (Rethorst et al., 2009; Krogh et al., 2011; Rebar et al., 2015; Kvam et al., 2016; Schuch et al., 2016; Vancampfort et al., 2017; Schuch et al., 2018), the impact of gender, age, population, and other factors on the results could not be determined.

**Table 7:** Top 10 cited references.

Rank	TLS^a^	Citations	Title	Year	Authors	Source	DOI
1	760	164	Exercise as a treatment for depression: A meta-analysis adjusting for publication bias	2016	Schuch Fb	J Psychiatr Res	10.1016/j.jpsychires.2016.02.023
2	623	120	Exercise treatment for depression: efficacy and dose response	2005	Dunn Al	Am J Prev Med	10.1016/j.amepre.2004.09.003
3	616	115	Exercise and pharmacotherapy in the treatment of major depressive disorder	2007	Blumenthal Ja	Psychosom Med	10.1097/psy.0b013e318148c19a
4	571	124	Exercise for depression	2013	Cooney Gm	Cochrane Db Syst Rev	10.1002/14651858.cd004366.pub6
5	506	84	Effects of exercise training on older patients with major depression	1999	Blumenthal Ja	Arch Intern Med	10.1001/archinte.159.19.2349
6	487	82	The antidepressive effects of exercise: a meta-analysis of randomized trials	2009	Rethorst Cd	Sports Med	10.2165/00007256-200939060-00004
7	411	89	Exercise as a treatment for depression: A meta-analysis	2016	Kvam S	J Affect Disorders	10.1016/j.jad.2016.03.063
8	400	56	The effect of exercise in clinically depressed adults: systematic review and meta-analysis of randomized controlled trials	2011	Krogh J	J Clin Psychiat	10.4088/jcp.08r04913blu
9	388	140	International physical activity questionnaire: 12-country reliability and validity	2003	Craig Cl	Med Sci Sport Exer	10.1249/01.MSS.0000078924.61453.FB
10	382	85	Physical activity and the prevention of depression: a systematic review of prospective studies	2013	Mammen G	Am J Prev Med	10.1016/j.amepre.2013.08.001

a TLS, total link strength.

**Table 8:** 8 meta-analysis studies.

Authors	Type of article	Studies	Participants	Studies and participant characteristics				Results	Main Findings
				Depression or anxiety	Gender	Age	Crowd		
Schuch FB 2016 (Hirsch, 2005)	Meta-analysis adjusting for publication bias	25	1,487	Depression	Females ranged from 17 to 100%.	18.4 to 76.4 years	Adults	Exercise had a large and significant effect on depression [SMD adjusted for publication bias = 1.11 (95%CI: 0.79–1.43)], with a failsafe number of 1,057.	Previous meta-analyses may have underestimated the benefits of exercise due to publication bias.
Schuch FB 2018 (Dunn et al., 2005)	Meta-Analysis of Prospective Cohort Studies	49	266,936	Depression	47% males	/	Adults and older	Compared with people with low levels of physical activity, those with high levels had lower odds of developing depression (adjusted odds ratio = 0.83, 95% CI = 0.79, 0.88; I^2^ = 0.00).	PA can confer protection against the emergence of depression regardless of age and geographical region.
Vancampfort D 2017 (Blumenthal et al., 2007)	Systematic review and meta-analysis	69	35,682	Depression	39.5% males	Mean age 43 years	/	People with severe mental illness were more sedentary than healthy controls (standard mean difference, SMD = 0.1; 95%CI: 0.0–0.2, *p* = 0.003, I^2^ = 37.1)	Translation of evidence-based interventions into routine care specifically aimed to reducing sedentary behavior and increasing physical activity is urgently.
Kvam et al. (2016)	Meta-analysis	23	97	Depression	/	/	/	Exercise as an adjunct to antidepressant medication yielded a moderate effect (g = −0.50) that trended toward significance.	Physical exercise is an effective intervention for depression. It also could be a viable adjunct treatment in combination with antidepressant -s.
Rethorst Cd 2009 (Schuch et al., 2016)	Meta-analysis	58	2,982	Depression	/	/	/	Participants in the exercise treatment had significantly lower depression scores than those receiving the control treatment (ES = −0.80, 95%CI =0.92, 0.67).	No significant differences between exercise and psychotherapy or antidepressant medications.
Krogh J 2011 (Rebar et al., 2015)	Systematic review and meta-analysis	13	13	Depression	/	/	Adults	Pooled results from these, the estimated beneficial effect of exercise was more modest (SMD, −0.19; 95% CI, −0.70 to 0.31) than the pooled result for all 13 studies, with no strong evidence of benefit.	A short-term effect of exercise on depression.
Rebar AL 2015 (Cooney et al., 2013)	Meta-analysis	92	4,310 for depression 10,755 for anxiety	Depression and anxiety	/	/	/	PA reduced depression by a medium effect, SMD = −0.50; 95% CI: −0.93 to −0.06, and anxiety by a small effect (SMD = −0.38; 95% CI: −0.66 to −0.11). No significant heterogeneity.	These findings represent a comprehensive body of high-quality evidence that physical activity reduces depression and anxietyin non-clinical populations.

### Analysis of keywords

**Figure 5:**
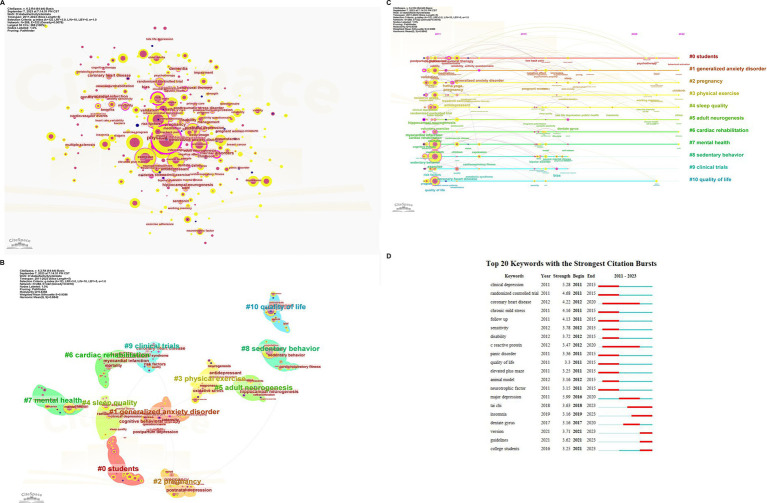
**(A)** Visualization of keywords. **(B)** Cluster analysis of keywoeds. **(C)** Timeline graph of cluster analysis. **(D)** Top 20 keywords with the most strongest bursts.

Furthermore, a visual map analysis of keywords was carried out using citespace (Figure 5A). As presented in Table 9, the top five keywords with the highest frequency were physical activity (652 times), symptoms (370 times), exercise (351 times), health (253 times), and Meta-analysis (245 times). In addition, the centrality, indicating the connectivity of nodes, of the following three keywords was greater than 0.5 and ranked amongst the top 3, namely physical activity (0.80), mental disorders (0.58), and risk (0.50). A higher centrality signifies a larger number of items connected through nodes, thereby reflecting the importance of nodes in the network. Generally, a centrality greater than 0.1 is regarded as significant.

**Table 9:** Top 10 keywords by frequency and centrality.

Rank	Centrality	Year	Keywords	Rank	Frequency	Year	Keywords
1	0.80	2011	Mental disorders	1	652	2011	Physical activity
2	0.58	2011	Physical activity	2	370	2011	Symptoms
3	0.50	2011	Risk	3	351	2011	Exercise
4	0.44	2011	Prevalence	4	253	2011	Health
5	0.40	2013	Generalized anxiety disorders	5	245	2011	Meta analysis
6	0.37	2011	Mortality	6	242	2011	Mental health
7	0.35	2011	Symptoms	7	204	2011	Quality of life
8	0.27	2011	Adult neurogenesis	8	189	2011	Prevalence
9	0.27	2011	Posttraumatic stress disorder	9	187	2011	Aerobic exercise
10	0.25	2011	Myocardial infarction	10	154	2011	Anxiety

Based on the keyword map, keyword clustering (Figure 5B) was performed to identify the main research content in this field. Modularity Q and Mean Silhouette are the basis for evaluating the rationality of scientific map clustering. Usually, if the Q value is greater than 0.3, the clustering structure is significant. If the S value is greater than 0.7, the clustering is convincing. In this clustering knowledge graph, the Q and S values were 0.8358 and 0.9386, respectively, suggesting an acceptable clustering. The top 11 keyword clustering labels were selected for analysis (Table 10). # 0 *students*, # 2 *pregnancy* belongs to the research object. # 1 *generalized anxiety disorder* is a disease. # 3 *physical exercise* and # 8 *sedentary behavior* are distinct lifestyles. # 4 *sleep quality*, # 6 *cardiac rehabilitation*, # 7 *mental health* and # 10 *quality of life* involve different observation indexes. # 5 *adult neurogenesis* is a physiological and pathological mechanism. # 9 *clinical trials* are research types. The keyword timeline diagram (Figure 5C) highlights the research progress of keyword nodes in each cluster.

**Table 10:** Top 11 largest clusters of keywords.

Cluster	Cluster label	Size	Silhouette	Average year
#0	Students	23	0.977	2014
#1	Generalized anxiety disorder	22	0.927	2015
#2	Pregnancy	21	0.913	2015
#3	Physical exercise	21	0.974	2014
#4	Sleep quality	18	0.973	2016
#5	Adult neurogenesis	18	0.962	2014
#6	Cardiac rehabilitation	17	0.928	2015
#7	Mental health	16	0.884	2014
#8	Sedentary behavior	16	0.967	2014
#9	Clinical trials	16	0.925	2013
#10	Quality of life	15	0.971	2013

Burst keywords with strong strength (Figure 5D) can reveal the research frontiers and development trends in a research field. In this study, significant research hotspots in this field in recent years were version (burst strength 3.71), tai chi (burst strength 3.63), guidelines (burst strength 3.62), college students (burst strength 3.25), insomnia (burst strength 3.16), and dentate gyrus (burst strength 3.16). According to the results of the burst keywords, the current hotspot research appears to be tai chi, focusing on a group of students based on the exploration mechanism of the dentate gyrus in the hippocampus.

## Discussion

Overall, the results of this study revealed the current status and trends in the field of PA on anxiety or depression. The research in this field has consistently expanded, demonstrating ongoing scholarly interest. Notably, the USA and Brazil have emerged as key countries in the field, with the latter depicting the closest and most central cooperation with other nations and the former conducting the highest number of research and the largest number of corresponding authors. Noteworthily, Harvard University remains a prolific contributor to this area of research. Among authors, Stubbs B was identified as the most prominent, with the highest number of published papers and H-index. A network of author cooperation centered on Stubbs B was also noted. Stanton R’s articles, published in 2020 (Stanton et al., 2020), garnered the highest total citations and were associated with the negative impact of Corona Virus Disease 2019 (COVID-19) on mental health in Australia. This finding may be ascribed to the particular attention paid by scholars on COVID-19-related articles. Among references considered in the final included studies, Schuch Fb’s article published in 2016 (Schuch et al., 2016) was the most frequently cited one. Their study determined that previous meta-analyses may underestimate the huge of exercise antidepressants due to publication bias.

According to the keyword frequency analysis (Table 9), meta-analysis (245 times) is a type of research that is of concern in this field. As the highest level of medical evidence, meta-analysis plays a fundamental role in clinical guidance. Josefsson et al. (2014) conducted a meta-analysis and demonstrated a significant overall effect of exercise intervention on depression. Thus, physical exercise is recommended for patients with depressive symptoms. At the same time, Zhang et al. (2023) conducted a network meta-analysis and found that resistance exercise 3–4 times a week for 30–60 min, lasting more than 6 weeks, exerted a substantial effect on the treatment and prevention of depression. Furthermore, Lin and Gao (2023) evaluated on the anxiety symptoms of college students and evinced the positive effect of PA on relieving anxiety, with aerobic exercise being potentially the optimal choice.

According to the keyword clustering map constructed from the research results, clusters #0 and #2 signaled that the field pays close attention to the anxiety and depression levels of students and pregnant women. With rapid economic expansion, the pressure faced by students is becoming increasingly heavy. However, most students grow up under the care and love of their families. As they enter the academic environment and confront the high pressure of learning and peer relationships, their mental health is affected (Slimmen et al., 2022). Therefore, it is vital to monitor students’ mental health and implement the necessary measures. Nonetheless, there are shortcomings in approving drug treatment regimens for young adults due to the unknown long-term risks on their future lives (You et al., 2021). At present, non-pharmacological interventions are preferred over drug therapy. Previous studies have concluded that low-intensity physical exercise can significantly enhance the mental health of college students, which can be attributed to the activation of potential targets in the brain (Zhu et al., 2020). Women experience physical and emotional fluctuations during pregnancy and childbirth. Pregnancy-related anxiety (PrA) is a special anxiety response characterized by fear and worry during pregnancy (Huizink et al., 2004), experienced by up to 14.4% (Poikkeus et al., 2006) of pregnant women. Additionally, postpartum depression is one of the most prevalent complications following childbirth. It comprises any depression women may experience from the beginning of their pregnancy until four weeks after delivery (Poyatos-León et al., 2017). According to a study undertaken by the U.S. Centers for Disease Control and Prevention (Bauman et al., 2020), approximately one in eight women develop postpartum depression. Hence, it is critical to timely identify pregnant and post-partum women with depression or anxiety disorders. Notwithstanding, it can have a devastating impact on mothers and children without timely intervention and, most importantly, exert a negative effect on mother-to-child relationships (Sanger et al., 2015). PA during pregnancy and postpartum is a viable strategy to achieve better mental health (Poyatos-León et al., 2017).

According to cluster #1, generalized anxiety disorder (GAD) is a prominent type of research disease in this field. With a global average lifetime prevalence of around 3.7% (Wittchen et al., 2002), GAD is among the most common types of anxiety disorders. Specifically, its prevalence is higher in women (Luo et al., 2019), who usually experience more severe symptoms of somatic anxiety (Steiner et al., 2005). A clinical study reported that exercise therapy is a feasible, safe, and well-tolerated treatment for GAD and could be a potential adjunctive therapy for sedentary women with GAD (Herring et al., 2012). According to cluster #8, this field focuses on sedentary behavior, which requires the least PA and leads to low energy consumption, similar to resting levels (Pate et al., 2009). It includes sitting for a series of purposes (such as work and travel) and screen-based activities, such as using computers, video games, and watching TV. Existing evidence suggests that sedentary behavior can increase the risk of cardiovascular disease (Katzmarzyk et al., 2009), osteoporosis (Warburton et al., 2006), and diabetes (Hu et al., 2003). Moreover, prior studies have established sedentary behavior as a risk factor for anxiety and depression (Huizink et al., 2004; Teychenne et al., 2010, 2015). In other words, sedentary behavior affects not only physical health but also mental health.

**Figure 6:**
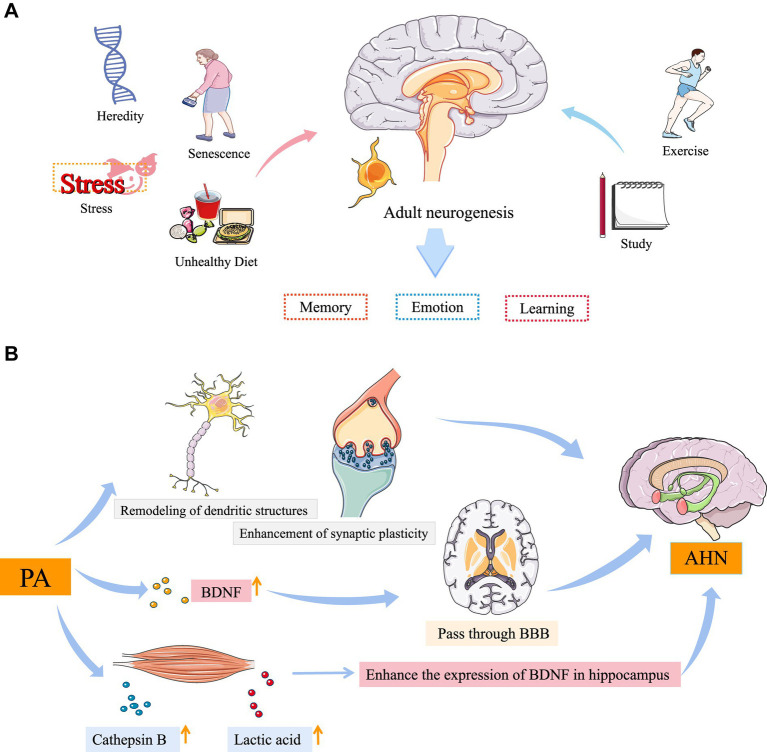
**(A)** Adult neurogenesis. **(B)** The possible mechanism underlying PA induced AHN. PA, physical activity; BNDF, brain-derived neurotrophic factor; BBB, blood–brain barrier; AHN, adult hippocampal neurogenesis. Images reproduced from Servier Medical Art (https://smart.servier.com/) under the terms of CC-BY 4.0.

Cluster #5 Adult neurogenesis (AN), the ability to produce new neurons from neural stem cells to adulthood, persists in specific regions of the brain throughout the life cycle of mammals, especially in the subgranular zone of the hippocampal dentate gyrus (DG) and the subventricular zone of the lateral ventricle (Ming and Song, 2011; Gage, 2019). Based on existing research, exercise (Schoenfeld and Swanson, 2021) and study (Gould et al., 1999) can promote adult neurogenesis. Conversely, stress (Hueston et al., 2017), heredity (Jacobs, 2002), senescence (Olariu et al., 2007), and unhealthy diet (Poulose et al., 2017) can inhibit AN (Figure 6A). According to the burst keyword dentate gyrus, we have noticed adult hippocampal neurogenesis (AHN) (Terreros-Roncal et al., 2021), which refers to the process by which neural stem cells synthesize new neurons in the hippocampus. It is universally acknowledged as a fundamental brain function, supporting endogenous regeneration and facilitating structural plasticity that aids the brain in adapting to environmental stressors (Mahar et al., 2014). Moreover, AHN plays a crucial role in learning, memory, and emotion regulation (Hill et al., 2015). To date, sufficient experimental evidence supports its role in governing depression and anxiety (Lim et al., 2021). Luo et al. (2023) claimed that exercise can effectively alleviate mental disorders, including anxiety and depression, and its neural mechanism can be attributed to improvements in AN. Nonetheless, the mechanism underlying PA-induced AN has not been determined so far. However, these important effects of PA in AN seem to be mainly mediated by neurotrophic factors including brain-derived neurotrophic factor (BDNF), insulin-like growth factor 1 (IGF-1), and vascular endothelial growth factor (VEGF) (Cotman et al., 2007). Nevertheless, whether these neurotrophins are the causes or the consequences of PA-induced neurogenesis and the mechanisms by which they mediate PA-induced neurogenesis remain to be determined. BDNF may be a convergent target of PA-induced peripheral factors in the brain (Gao et al., 2023). To begin, numerous peripheral factors are released, among which BDNF shows the most significant increase in the brain. Moreover, BDNF is a positive regulator of mature AN (Liu and Nusslock, 2018). Peripheral BDNF can pass through blood–brain barrier (BBB) (Pan et al., 1998). Finality, PA can up-regulate the expression of cathepsin B in muscles and drive the accumulation of metabolite lactic acid, both of which can cross the BBB and enhance the expression levels of BDNF in the hippocampus (Moon et al., 2016; El Hayek et al., 2019). Furthermore, PA can induce structural and functional changes, including an increase in adult neurogenesis (van Praag et al., 1999), remodeling of dendritic structures (Eadie et al., 2005), and enhancement of synaptic plasticity in the hippocampus (van Praag et al., 1999), suggesting a biological basis for PA in protecting the brain against stress (Figure 6B). A prior study (Li et al., 2019) has suggested that PA may rescue impaired neuronal plasticity by regulating neurotrophic factors and adjusting the adaptive response of the hypothalamic–pituitary–adrenal axis to stress.

According to the results of the burst keywords, recent studies focused on Tai Chi, a traditional Chinese sports that is currently classified as mind–body exercise (MBE, exercise aiming to improve participants’ mind–body coordination and awareness by having them practice a series of controlled movements that focus on interactions among the brain, body, mind, and behavior, such as Tai Chi, yoga, and dance, etc.) (Huang et al., 2022). It has been extensively applied for the prevention and treatment of various diseases, such as cancer (Song et al., 2020), knee osteoarthritis (Zhang et al., 2023), mild cognitive impairment (Zhang et al., 2019), and so on. It is worthwhile emphasizing that a meta-analysis has demonstrated that Tai Chi is more effective than non-mindfulness exercises in reducing anxiety and depression levels and improving general mental health (Yin et al., 2023). Moreover, Tai Chi possesses the advantage of minimal equipment requirements and is not limited by location or facilities.

According to the keywords, students and pregnant women were identified as groups of concern. Similarly, AN was established as a physiological and pathological mechanism of interest. Sedentary behavior was the lifestyle of concern, while GAD is the type of disease of concern. Finally, Tai Chi is the type of PA of interest.

### Limitation

Reviewing previous studies, there are bibliometric analyses focusing on exercise and depression in teenagers (You et al., 2021) and college students nearly 20 years ago (Ai et al., 2023), as well as on the 50 most-cited articles (Zhai and Xu, 2023). This study did not impose restrictions on time and group. However, there are inherent limitations associated with this study that cannot be overlooked. Firstly, due to the shortcomings of the current bibliometric analysis software, it is challenging to analyze the data from multiple databases, and thus, this study exclusively screened articles from the WOSCC database. In addition, to mitigate the influence of confounding factors in the abstract, articles were searched by title, potentially introducing bias in the search strategy. Finally, our analysis solely included articles and reviews published in English and excluded abstracts, conferences, or books. Therefore, the results of the study may lack comprehensive coverage. On the other hand, while we analyzed the meta-analysis studies in cited literature and references, the extracted information did not provide valuable information on the impact of gender, age, population, and other factors on the results of the study, potentially impacting the discussion results cited in the review and meta-analysis.

## Conclusion

In this study, the bibliometric analysis provided information regarding emerging trends about the field of PA on anxiety or depression. Although this work has some limitations, the results may be useful as a basis for helping patients with anxiety or depression and as a foundation for further research in this field. Based on our results, the field of PA on anxiety or depression demonstrating ongoing scholarly interest. Students and pregnant women, adult neurogenesis, and Tai Chi were the prominent keywords. For clinicians, they can consider to recommend patients to perform mind–body exercise for improving mood, especially for students and pregnant women who use medications with cautions. Additionally, future researchers can explore the mind–body exercise and its impact on anxiety or depression, PA and anxiety or depression in specific populations, and adult neurogenesis of various exercise in anxiety or depression.

## Data availability statement

The original contributions presented in the study are included in the article, further inquiries can be directed to the corresponding author.

## Author contributions

X-YZ: Writing – original draft. FY: Supervision, Writing – review & editing. Z-HY: Supervision, Writing – review & editing. Y-QL: Data curation, Writing – original draft. Q-NB: Data curation, Writing – original draft. M-ZX: Investigation, Writing – original draft. Z-HC: Visualization, Writing – original draft. W-QZ: Visualization, Writing – original draft. K-XW: Investigation, Writing – original draft. JY: Investigation, Writing – original draft. F-RL: Writing – review & editing.
